# The Evolutionary Traceability of a Protein

**DOI:** 10.1093/gbe/evz008

**Published:** 2019-01-15

**Authors:** Arpit Jain, Dominik Perisa, Fabian Fliedner, Arndt von Haeseler, Ingo Ebersberger

**Affiliations:** 1 Applied Bioinformatics Group, Institute of Cell Biology & Neuroscience, Goethe University, Frankfurt, Germany; 2 Center for Integrative Bioinformatics Vienna, Max F. Perutz Laboratories, University of Vienna, Medical University Vienna, Austria; 3 Bioinformatics and Computational Biology, Faculty of Computer Science, University of Vienna, Austria; 4 Senckenberg Biodiversity and Climate Research Center (BiK-F), Frankfurt, Germany; 5 LOEWE Centre for Translational Biodiversity Genomics (LOEWE-TBG), Frankfurt, Germany

**Keywords:** ortholog search, twilight zone, sequence evolution, phylogenetic profile, metabolic pathway, LUCA

## Abstract

Orthologs document the evolution of genes and metabolic capacities encoded in extant and ancient genomes. However, the similarity between orthologs decays with time, and ultimately it becomes insufficient to infer common ancestry. This leaves ancient gene set reconstructions incomplete and distorted to an unknown extent. Here we introduce the “evolutionary traceability” as a measure that quantifies, for each protein, the evolutionary distance beyond which the sensitivity of the ortholog search becomes limiting. Using yeast, we show that genes that were thought to date back to the last universal common ancestor are of high traceability. Their functions mostly involve catalysis, ion transport, and ribonucleoprotein complex assembly. In turn, the fraction of yeast genes whose traceability is not sufficient to infer their presence in last universal common ancestor is enriched for regulatory functions. Computing the traceabilities of genes that have been experimentally characterized as being essential for a self-replicating cell reveals that many of the genes that lack orthologs outside bacteria have low traceability. This leaves open whether their orthologs in the eukaryotic and archaeal domains have been overlooked. Looking at the example of REC8, a protein essential for chromosome cohesion, we demonstrate how a traceability-informed adjustment of the search sensitivity identifies hitherto missed orthologs in the fast-evolving microsporidia. Taken together, the evolutionary traceability helps to differentiate between true absence and nondetection of orthologs, and thus improves our understanding about the evolutionary conservation of functional protein networks. “protTrace,” a software tool for computing evolutionary traceability, is freely available at https://github.com/BIONF/protTrace.git; last accessed February 10, 2019.

## Introduction

The question “How old is a gene?” is fundamental in functional and evolutionary genetics (Capra et al. 2013). The age of a gene is tightly linked to many of its functional properties. Proteins encoded by old genes tend to evolve slightly slower than younger genes (Alba and Castresana 2005, 2007; Wolf et al. 2009); however, as seen in Elhaik et al. (2006), they are expressed in more tissues (Freilich et al. 2005), are more central in protein–protein-interaction networks (Kim and Marcotte 2008), and seem involved in more complex regulatory networks (Warnefors and Eyre-Walker 2011). It, thus, comes as little surprise that gene age is a good proxy for the essentiality of the encoded protein’s function (Gustafson et al. 2006; Hwang et al. 2009) and that older genes are more often associated with human diseases (Domazet-Loso and Tautz 2008; Cai et al. 2009; Maxwell et al. 2014).

Assessing the age of a gene, however, is not trivial (Capra et al. 2013), as none of the above characteristics can be attributed exclusively to old genes (Wolf et al. 2009). Instead, age estimates are typically derived from interpreting, for each gene, the phylogenetic distribution of its orthologs (Mirkin et al. 2003). Under the simplifying assumption that genes are only transferred vertically from ancestor to descendent, the last common ancestor of the two most distantly related species in a phylogeny that harbors an ortholog approximates the minimal age of the corresponding gene (see, however, Doolittle 1999; Gogarten et al. 2002). Genes of the same age can then be summarized in phylostrata (Domazet-Loso et al. 2007), which inform about the lineage-specific evolution of gene repertoires (Ebersberger et al. 2014), and allow the correlation of genetic innovation with major changes during organismal evolution (Slamovits et al. 2004; Domazet-Loso et al. 2007; Sestak and Domazet-Loso 2015). The oldest layers in the phylostrata comprise the genes whose orthologs span a considerable range or even the full diversity of contemporary life. These genes are likely to hold a key position in the metabolic network, and their widespread phylogenetic distribution implies that a loss is detrimental for survival (Mushegian and Koonin 1996). In particular, those genes that can be traced back to the last universal common ancestor (LUCA) (Woese 1998; Goldman et al. 2013) have been used to deduce a molecular scaffold essential for organismic life (Koonin 2003).

The design of artificial life both challenges and complements the evolutionary inferences of a universal genetic repertoire common to—and necessary for—all living organisms (reviewed by Rancati et al. [2018]). Only recently, 473 genes from *Mycoplasma mycoides* were determined as the minimal gene (MG) set required, under the most favorable conditions (Koonin 2003), for a self-replicating cell (Hutchison et al. 2016). Many of these genes have detectable homologs only in bacteria or even only in the genus *Mycoplasma* (Hutchison et al. 2016), suggesting an evolutionarily recent origin. This is at odds with the expectation that essential genes have a wide phylogenetic spread (Jordan et al. 2002). Instead, it seems to indicate that also essential genes are subject to evolutionary change (Rancati et al. 2018). For example, a gene responsible for an essential function can be replaced by an unrelated, yet functionally equivalent gene a process called nonorthologous gene displacement (Koonin et al. 1996; Phadnis et al. 2012; Huynen et al. 2013; Kachroo et al. 2015; Zallot et al. 2017). Alternatively, genes that are essential in one organism may not be essential in another (Liao and Zhang 2008; Koo et al. 2017). This is, for example, because a closely related paralog can complement its function, because its metabolic network has become more robust by evolving redundancy, or because the metabolic network was rewired to bypass the essentiality of individual proteins (Kim et al. 2010; Rancati et al. 2018). In any case, this would imply that the *M.mycoides* MG set represents only a minor step toward unraveling the universal building plan of organismic life.

However, sequence similarity used to identify orthologs in present-day gene sets decays with time (Dayhoff 1978). Ultimately, a twilight zone (Doolittle 1981) is hit where two related proteins are no longer similar enough to infer common ancestry (Dayhoff 1978; Rost 1999). The time to reach the twilight zone varies between proteins and depends on their sequence composition as well as their substitution rate (Dayhoff 1978) but not on their essentiality (Hurst and Smith 1999; Hirsh and Fraser 2001). This links the accuracy of the gene age assessment to the sensitivity of the ortholog identification methods. This issue was first raised by Elhaik et al. (2006) who used a simulation-based approach to show that the sensitivity of BlastN (Altschul et al. 1997) can be a limiting factor in the identification of homologs when evolutionary distances are large. As a consequence, the sharing of essential genes between distantly related or fast-evolving species will be overlooked, and gene ages will be underestimated (Elhaik et al. 2006; Luz et al. 2006; Moyers and Zhang 2015, 2016, 2017). The risk of misinterpreting the evolutionary past is therefore high (Liebeskind et al. 2016; Martín-Durán et al. 2017). Using more sensitive search algorithms that are dedicated to a remote homolog detection (e.g., PSI-Blast [Altschul et al. 1997] or HHsearch [Soding 2005], for an overview see Chen et al. [2018]) can ameliorate this issue, in principle. However, these algorithms do not differentiate between orthologs and paralogs. In the context of inferring the evolutionary history of a particular gene they must, thus, be used with caution. They should only then be applied when sufficient evidence exists that an ortholog might have diverged to an extent that it is no longer detectable by a conventional ortholog search tool. Individual approaches exist that aim at delineating, for a given protein, the evolutionary distance beyond which orthologs no longer share a significant sequence similarity (Moyers and Zhang 2016); standardized solutions that have been cast into a dedicated software are not yet at hand.

Here, we introduce for each protein its (*evolutionary) traceability*. This measure informs over what evolutionary distances the sequence similarities between orthologs should be still high enough to allow their detection with standard ortholog search software. Using the yeast gene set as an example, we find that genes with a consistently high traceability index across species are enriched for catalytic functions in the cell metabolism. The subset of yeast genes whose evolutionary origins have been dated back to LUCA almost entirely belongs to this group. For a substantial fraction of the yeast genes however, among them many with essential functions, the traceability index decays quickly. For these genes, the sensitivity of a standard ortholog search can become a limiting factor in more distantly related species. These findings suggest a new interpretation of the evolutionary conservation pattern of the MG set. The vast majority of the MG-set proteins that appear confined to bacteria show low traceabilities, which indicates that the sensitivity of the ortholog search becomes limiting in species other than bacteria. Thus, there is a high chance that archaeal or eukaryotic orthologs exist but have been overlooked. Looking at the example of yeast Rec8, a protein essential for recombination, we show how a traceability-informed increase of the ortholog search sensitivity can lead to the identification of hitherto overlooked representatives in fast-evolving species.

## Materials and Methods

### Data Sets

Our analyses are based on 232 species representing the three domains of life (supplementary table S1, Supplementary Material online). The taxonomic tree for these species was obtained from NCBI CommonTree (https://www.ncbi.nlm.nih.gov/Taxonomy/CommonTree/wwwcmt.cgi; Last accessed February 10 2019).

The LUCA gene sets (1,203 genes) were downloaded from LUCApedia (Goldman et al. 2013), a database consisting of all LUCA gene sets proposed by different studies. The essential genes set (1,110 genes) for *Saccharomycescerevisiae* was obtained from database of essential genes (Luo et al. 2014). The LUCA genes and the essential genes are listed in supplementary table S3, Supplementary Material online. Aligned orthologous groups from the sensu stricto group of yeast species were retrieved from http://www.saccharomycessensustricto.org/current/aligns/coding_allfiles.fasta.tgz last accessed February 10, 2019 (Scannell et al. 2011).

### Compilation of Orthologous Groups

First, orthologs for the seed protein are retrieved from the corresponding ortholog group provided by the OMA database (Altenhoff et al. 2015). We then extend the OMA ortholog group with sequences from a collection of 232 species (supplementary table S1, Supplementary Material online) using HaMStR (Ebersberger et al. 2009), a profile hidden Markov model (pHMM)-based ortholog search tool. HaMStR was run with the following parameters: *-strict*, *-checkCoorthologsRef*, *-hit_limit = 1*, and *-representative*. For query proteins without orthologs in the OMA database, we directly perform a targeted ortholog search using HaMStR-OneSeq (https://github.com/BIONF/HaMStR; last accessed February 10, 2019; Ebersberger et al. 2014) in the gene sets of 232 species. HaMStR-OneSeq is an extended version of HaMStR that compiles in an iterative procedure an initial core-ortholog set for pHMM training. Once the training is completed, a final ortholog search in all taxa concludes the procedure. HaMStR-OneSeq is run with the following parameters: *-coreOrth = 5*, *-minDist=genus*, *-maxDist=superkingdom*, *-checkCoorthologsRef*, *-strict*, and *-rep*. Alternatively, we used ortholog groups provided by OrthoDB (Zdobnov et al. 2017) for parameterizing the evolutionary models.

### Maximum Likelihood Distance Estimation

We computed pairwise maximum likelihood (ML) distances between proteins using TreePuzzle v5.225 (Schmidt et al. 2002). To arrive at an average ML genetic distance between any pair of species, we extracted and aligned all pairwise orthologs for the two species from the OMA database (Altenhoff et al. 2015). In the case of 1:many ortholog groups, we considered all induced pairwise orthology relationships. The alignments were then concatenated and served as input for TreePuzzle to compute an average ML distance. The procedure was repeated for all species pairs in the reference tree to obtain an all-against-all ML distance matrix.

### Annotation of Pfam Domains

We annotated Pfam (Finn et al. 2016) domains using *hmmscan* (Finn et al. 2011) with parameters *–notextw* and *-E 0.01*.

### Prediction of Subcellular Localization

We predicted the subcellular localization of the yeast proteins following the approach of Sojo et al. (2016). Precisely, we annotated transmembrane domains with *tmhmm* v2.0 (Sonnhammer et al. 1998) to differentiate between membrane and water-soluble proteins. For the fraction of water-soluble proteins, we subsequently used *signalp* v4.1 (Petersen et al. 2011) to distinguish them into extracellular proteins and intracellular proteins.

### Gene Ontology Term Enrichment Analysis

We searched for Gene Ontology (GO) terms enriched in a set of yeast proteins with GOrilla (Eden et al. 2009). The entire gene set of *S.cerevisiae* served as the background set. An *E*-value cutoff of 10^−3^ was applied. Significantly enriched GO terms were then visualized using Revigo (Supek et al. 2011).

### Phylogenetic Analysis

The domain annotation of REC8 in yeast (*S. cerevisiae*) revealed the presence of a Rad21_REC8_N domain (PF04825). Using the Rad21_REC8_N profile HMM obtained from Pfam (Finn et al. 2016), we searched with *hmmsearch* (Finn et al. 2011) for proteins harboring this domain in the gene sets of ten microsporidia (*Encephalitozoon cuniculi*, *Encephalitozoon hellem*, *Encephalitozoon intestinalis*, *Antonospora locustae*, *Nosema ceranae*, *Enterocytozoon bieneusi*, *Edhazardia aedis*, *Anncaliia algerae*, *Vittaforma corneae*, and *Nematocida parisii*) and of yeast. The search in yeast resulted in a second protein, MCD1/SCC1, also containing the Rad21_REC8_N domain. We then retrieved REC8 and MCD1/SCC1 orthologs from training data used for the traceability calculation in the following fungal and outgroup species—*Ashbya gossypii*, *Yarrowia lipolytica*, *Fusarium graminearum*, *Verticillium dahliae*, *Phanerochaete chrysogenum*, *Schizosaccharomyces pombe*, *Tremella mesenterica*, *Ustilago maydis*, *Heterobasidion irregulare*, *Phycomyces blakesleeanus*, *Batrachochytrium dendrobaditis*, *Capsaspora owczarzaki*, *Monosiga brevicollis, Amphimedon queenslandica*, *Nematostella vectensis*, *Drosophila melanogaster*, and *Homo sapiens*. Because both OMA and HaMStR found no orthologs to yeast REC8 in animals, we complemented the data with the *H. sapiens* REC8 protein (NCBI accession: NP_001041670), and its InParanoid (Ostlund et al. 2010) orthologs from *Gasterosteus aculeatus*, and *Daphnia pulex*. All sequences were aligned with MAFFT v7.304 using the option *L-INS-i*. From the resulting multiple sequence alignment (MSA), we computed an ML tree with 100 bootstraps using RAxML v8 (Stamatakis 2014), modeling the substitution process with *PROTGAMMALG*, the best model obtained from ProtTest v3 (Abascal et al. 2005). Tree topology testing was performed using the routines implemented in RAxML. Pfam domain architecture display on a phylogenetic tree was done with doMosaics (Moore et al. 2014).

### Data Availability

All data that support the finding of this study are available via figshare: https://figshare.com/projects/yeast_traceability_metadata/56348; last accessed February 10, 2019.

## Results and Discussion

### protTrace: A Simulation-Based Workflow to Estimate the Evolutionary Traceability of a Protein

**Fig. 1.**
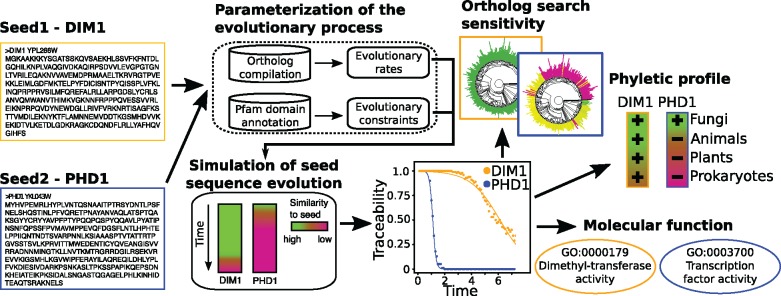
—Workflow to assess the evolutionary traceability of a protein. We show as examples two yeast proteins, PHD 1(blue) and DIM1 (yellow). For each seed protein, we use a simulation-based approach to infer its traceability, TI(*t*), that is defined on the interval [0, 1]. From its traceability graph and the evolutionary distance to any target species, the traceability index of the seed in the target species can be extracted. Relating this information to 1) a species tree highlights taxa where the ortholog search sensitivity becomes limiting (red clades), 2) phylogenetic profiles identifies cases where orthologs might have been overlooked, and 3) the gene ontology identifies molecular functions that coincide with low traceability.

protTrace determines for a user-defined protein--the seed protein—ts traceability as a function of evolutionary time. The procedure comprises four main steps—1) parameterization of a site-specific evolutionary model, 2) simulation of protein sequence evolution, 3) the calculation of the traceability, and optionally 4) the display of the traceabilities on a reference tree. The general workflow is represented in figure 1 and more detailed information is provided in supplementary figure S1*A*, Supplementary Material online, and in the software documentation on GitHub.

#### Step 1—Parameterization of the Evolutionary Process

First, protTrace infers the evolutionary characteristics of the seed-protein. We compile a group of orthologs, *O*_seed_, for the seed-protein. protTrace facilitates the use of precompiled orthologs from OMA (Altenhoff et al. 2015), InParanoid (Ostlund et al. 2010), and OrthoDB (Zdobnov et al. 2017). Optionally, a targeted ortholog search with HaMStR (Ebersberger et al. 2009) can be employed. In the next step, the orthologous sequences are aligned with MAFFT v7.304 (Katoh and Toh 2008), and an ML tree, *T*_seed_, is computed with RAxML v8 (Stamatakis 2014). The resulting tree and the MSA are then used to determine the evolutionary parameters of the proteins as follows. A maximum parsimony algorithm infers the seed-protein-specific insertion and deletion (indel) rates (supplementary fig. S1*B*, Supplementary Material online). Note, we preferred the parsimony algorithm over more elaborated methods to infer the indel rate, such as Sparta (Levy Karin et al. 2015) or SpartaABC (Ashkenazy et al. 2017), for performance reasons. The run times for these programs can be in the range of hours for alignments of hundred or more sequences, in contrast to seconds for the parsimony algorithm. A comparison of indel rates estimated once with the parsimony algorithm and once with Sparta revealed rates in the same range (supplementary fig. S2, Supplementary Material online). The distribution of the insertion rates, in this yeast protein set example, is shown in supplementary figure S3*A*, Supplementary Material online. Finally, the indel lengths of one most parsimonious solution are used for estimating *p*, the parameter of the geometric indel length distribution. With *hmmscan* (Finn et al. 2015) (parameters: *–notextw* and *-E 0.01*) we identify regions in the seed protein representing Pfam-A (Finn et al. 2016) domains. From the corresponding pHMMs of the Pfam domains, we extract the information for a site-specific domain constraint on the evolutionary process (Koestler et al. 2012).

In a phylogenomic setting, the evolutionary parameters are inferred for many seed-proteins, for example, all proteins encoded in a species’ genome. To account for different absolute substitution rates between the individual seed-proteins, we introduce the rate scaling factor *κ*_seed_ (eq. 1). We compute *κ*_seed_ for each seed-protein as

κseed=Mediani≠jdseedi,jd-speciesi,j,1

where *d*_seed_(*i, j*) is the ML distance between the orthologs in *O*_seed_ for species *i* and *j*, and ${\overset{-}{d}}_{\mathrm{species}}(i,j)$ is the average ML distance across all pairwise orthologs for the two species *i* and *j*. In the context of this study, we used the pairwise ortholog assignments from OMA (Altenhoff et al. 2015), but any other assignment method can be applied, in principle. If $\frac{d_{\text{seed}}\left(i,j\right)}{{\overset{-}{d}}_{\text{species}}\left(i,j\right)}>1$, then the seed protein evolves for species pair (*i, j*) faster than the average protein in OMA, otherwise (<1) slower. *κ*_seed_ is then the median of the ratios inferred from all species pairs *i, j* in *O*_seed_. Supplementary figure S3*B*, Supplementary Material online, shows the distribution of *κ*_seed_ exemplarily across all yeast proteins.

#### Steps 2 and 3—Simulation of Protein Sequence Evolution and Calculation of the Traceability Curve

Once the evolutionary model is fully parameterized, protTrace uses REvolver (Koestler et al. 2012) to simulate the evolution of the seed protein in time steps of 0.1 substitutions per site. In brief, REvolver takes the seed protein and the substitution model together with the substitution- and indel rates as input. As the main feature that distinguishes REvolver from other simulators (e.g., ROSE [Stoye et al. 1998] or Indel-SeqGen [Strope et al. 2007]), the program can take, the Pfam domain annotation for the seed protein into account. The pHMMs of Pfam domains are derived from curated alignments of homologous proteins or protein domains. Thus, they capture information regarding which sequence sites remained conserved over time and where in a sequence insertions or deletions are more likely to occur. In essence, they describe constraints on the evolutionary process acting on these sequences. REvolver uses this information for automatically parameterizing site-specific models of sequence evolution for the seed protein. After each step, the simulated sequence serves as a query for a BlastP (Altschul et al. 1997) search with default settings against the full protein set of the species the seed-protein was derived from (seed species). We use BlastP for this search, because this tool, or a comparable database search algorithm using local sequence alignments, is implemented in many popular ortholog search tools (e.g., OMA [Altenhoff et al. 2015], InParanoid [Remm et al. 2001], OrthoDB [Zdobnov et al. 2017], or orthoMCL [Li et al. 2003]). If the seed-protein sequence is identified as one of the top five hits, the success is marked with a “1,” otherwise a “0” is noted. Repeating the simulation 100 times yields for each time step a fraction of successes. To determine the traceability index of the seed protein as a function of time, TI(*t*), we fit the inverse of a nonlinear least square logistic growth curve to these data (eq. 2) using the nonlinear least square (*nls*) package in R:

TIt=1-N0erκseedt1+N0erκseedt-1.(2)

We estimate the parameters *N*_0_, and *r*, the rate change of traceability, from the data. For a given evolutionary time *t*_1_, the TI ranges between 0 and 1. A TI(*t*_1_) of 0 indicates that in none of the 100 simulations the simulated sequence that was evolved up to time *t*_1_ had its seed protein within the top five BlastP hits. An ortholog search based on sequence similarity is bound to fail. In turn, a TI(*t*_1_) of 1 indicates that in each of the 100 simulations the seed protein was within the top five BlastP hits. The sequence similarity should be, thus, sufficiently high for an ortholog detection.

#### Step 4—Tree Display

To provide for each seed protein an intuitive overview, protTrace can display the traceability information along a species phylogeny (supplementary fig. S4, Supplementary Material online). Here, the color of the leaf labels indicates the traceability index of the seed-protein in the respective species.

#### Implementation

protTrace is implemented as a command-line tool in Python 2.7, and helper scripts are written in Java, Perl, and R (R Core Team 2018). It runs on the three main operating systems, Linux, MacOS, and Windows, although we have tested protTrace only on Linux and MacOS. protTrace is distributed as open source according to the GNU-GPL3.0 license via GitHub (https://github.com/BIONF/protTrace; last accessed February 10, 2019), and an accompanying WIKI is provided with the software.

### The Evolutionary Traceability of the Yeast Gene Set

Yeast (*S.cerevisiae*), as a genetically and functionally well-characterized model organism, provides an excellent starting point for exemplifying the concept and implications of protein traceability (fig. 1). We compiled for each of the 6,352 yeast proteins its ortholog group, and then used these data to estimate the scaling factor, κ, and the indel rate. Supplementary Figure S5, Supplementary Material online, gives an overview of the ortholog group sizes. Both scaling factors and indel rates are negatively correlated with the ortholog group size (supplementary fig. S6, Supplementary Material online). This indicates that methodological issues, such as difficulties in aligning distantly related sequences in large and phylogenetically diverse ortholog groups, do not interfere with the evolutionary rate estimation. We then used protTrace to determine the traceability indices, TI(*t*), for 6,352 yeast proteins in 232 target species representing all three domains of life (supplementary tables S1 and S2, Supplementary Material online). For the 1,381 yeast proteins with <3 sequences, we used as default the mean of the indel rate distribution across the entire protein set (0.08) (supplementary fig. S3*A*, Supplementary Material online). In addition, we set the parameter *p* of the geometric distribution, from which we draw the length of insertions and deletions in regions not covered by a Pfam domain, to 0.25, the default value implemented into REvolver (see supplementary fig. S7, Supplementary Material online, for further discussion). If no ortholog was detected for a seed-protein, we used the mean of the scaling factor distribution across all yeast proteins (*κ*_mean_*=*1.57) as the default value (supplementary fig. S3*B*, Supplementary Material online). The result of the traceability computation for these proteins is summarized in supplementary figure S8, Supplementary Material online. Orthologous groups based on OMA and complemented with HaMStR (see Materials and Methods), or compiled with OrthoDB obtained highly correlated results (*r* = 0.92; see supplementary fig. S9, Supplementary Material online). The choice of the ortholog search method has therefore little impact on the traceability estimate, and we used the traceability indices obtained from the OMA/HaMStR approach for the remainder of the analysis. Likewise, there was virtually no impact on the traceability estimates if we recruited the orthologs for estimating the evolutionary parameters from species across the entire tree of life or only from fungal species (supplementary fig. S10, Supplementary Material online). This indicates that already the fungal orthologs are sufficient to capture the long-term evolutionary characteristics of the seed proteins.

**Fig. 2.**
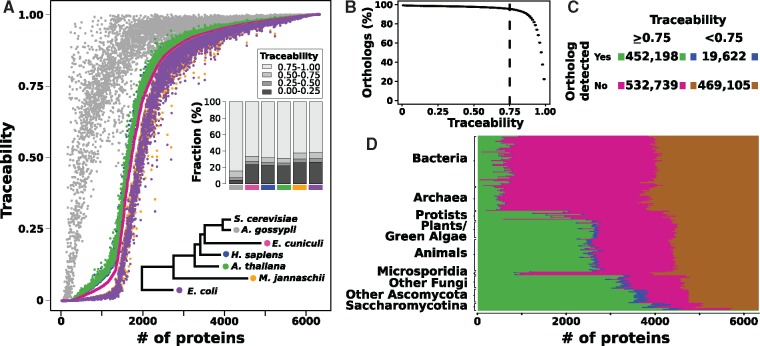
—The evolutionary traceability of yeast proteins. (*A*) Traceability indices for 6,352 yeast proteins in *Ashbya gossypii* (Fungi), *Encephalitozoon cuniculi* (Microsporidia), *Homo sapiens* (Metazoa), *Arabidopsis thaliana* (Viridiplantae), *Methanocaldococcus jannaschii* (Archaea), and *Escherichia coli* (Bacteria). Proteins are ordered according to their traceability index in *E. cuniculi*. The inlay shows a stacked bar plot providing, for each species, the fraction of proteins in each of the four traceability bins. The color code identifying the individual species is specified in the phylogenetic tree. (*B*) Cumulative distribution of the detected yeast orthologs relative to the protein's traceability index. Of the detected orthologs, 95% coincide with a traceability index of 0.75 or above in the respective species (hatched line). (*C*) Relation between results of the ortholog search and protein traceability. (*D*) Per-species results with the color code following (*C*).

Figure 2*A* displays the traceabilities of the yeast proteins for four eukaryotes, one archaeon, and one bacterium. For 2,040 proteins, the traceability indices decrease only very slowly with increasing evolutionary distance between yeast and the target species (*TI*(*t*) ≥ 0.95 for all target species). As we cover the full phylogenetic diversity in the tree of life, the rate and pattern of evolutionary sequence change for these proteins should not hinder ortholog detection in any extant species. For the remaining 4,312 proteins, phylogenetic distance and the evolutionary rate of the target species jointly determine protein traceability. When moving from the closely related fungus, *A. gossypii*, to archaea and bacteria, the number of proteins with a traceability of 0.0 increases by an order of magnitude (fig. 2*A*). Likewise, the traceability indices are considerably smaller in the microsporidium *E. cuniculi*, an obligate intracellular parasite closely related to fungi (Thomarat et al. 2004), than in human and *Arabidopsis* that belong to different kingdoms. This is an effect of the extraordinarily high substitution rate in the microsporidian lineage, which is among the highest across all eukaryotes (Slamovits et al. 2004).

We next calibrated the traceability index. It should inform in real data about the evolutionary distance beyond which orthologs are too diverged to be detected with BlastP-based ortholog search tools For the 6,352 yeast proteins, we searched for orthologs in the 232 target species, and we tabulated the number of yeast-species pairs in which at least one ortholog was found. In 95% of the cases where an ortholog was detected, the traceability was at least 0.75 (fig. 2*B*). Thus, we conclude, when the traceability is below 0.75, an ortholog search will probably fail. If an ortholog exists, it has likely diverged beyond recognition. Based on the TI threshold of 0.75, we distinguish two scenarios for the cases where no ortholog was identified (fig. 2*C*). For the 53% of cases where the TI is larger or equal to 0.75, we conclude that the ortholog is absent, as we should be able to detect it otherwise. For the remaining 47%, the TIs do not reach the threshold of 0.75, and such cases occur in almost all target species (fig. 2*D*). In other words, in almost half of the cases where we do not find an ortholog for a yeast protein, we cannot distinguish, without further evidence, between true absence and insufficient search sensitivity.

We are aware of one study that used a simulation-based approach to predict for yeast genes the maximal evolutionary distances in which BlastP still finds a homolog gene (Moyers and Zhang 2016). In this study, the authors inferred their constraints on the evolutionary process for each yeast protein from the alignment of orthologs of five sensu stricto yeast species. Because Moyers and Zhang (2016) did not link their findings to the actual phylogenetic profiles of the yeast proteins, comparing their results with our study is hard. We therefore reproduced their analysis in part. Moyers and Zhang (2016) used site-specific substitution rate scaling factors inferred with TreePuzzle (Schmidt et al. 2002) as information to constrain the evolutionary process. We recreated these constraint vectors, once with the original approach by Moyers and Zhang (2016) using the five sensu stricto yeast sequences, and once with an alignment using orthologs selected from the full diversity of fungi. This revealed that the phylogenetic diversity of the input alignment has a strong effect on the constraint pattern. When using the sensu stricto yeast orthologs, on average 80% of the alignment sites are assigned a relative rate of zero. Such positions remain unchanged in the course of simulated evolution. In contrast, when using the phylogenetically diverse training data, on average only about 15% of the alignment sites get assigned a relative rate of zero (supplementary fig. S11, Supplementary Material online). Thus, the evolutionary constraint information—and as a consequence the traceability of the protein over time (supplementary fig. S12, Supplementary Material online)—changes with the underlying training data. In the particular case of the simulated yeast protein evolution (Moyers and Zhang 2016), it appears that the use of the closely related yeast sequences for inferring the site-specific puts a too harsh constraint on the evolutionary process (supplementary fig. S11, Supplementary Material online). Using our terminology, this is bound to result in an overestimated traceability, an aspect that the authors have noted themselves (Moyers and Zhang 2017).

### Unobserved Domain Constraints Result in Underestimated Traceabilities

**Fig. 3.**
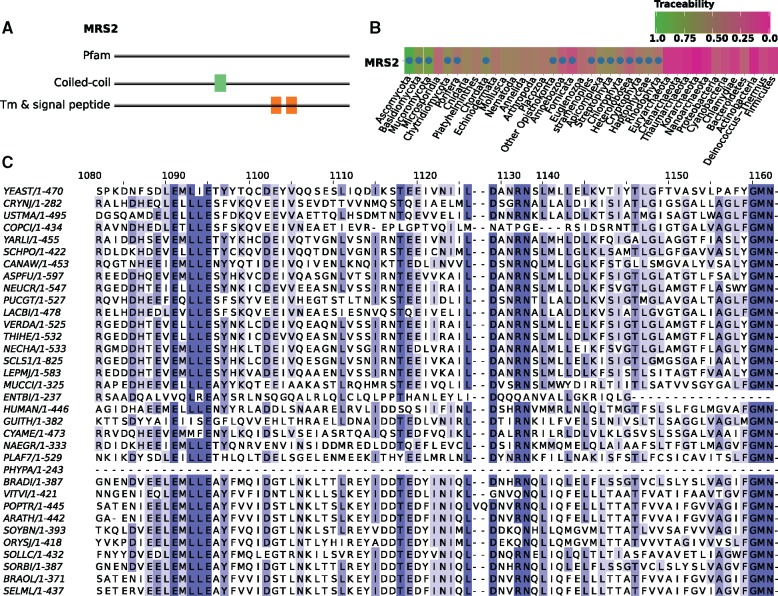
—Missing information about domain constraints results in underestimated traceabilities: the yeast mitochondrial inner membrane Mg^2+^ transporter MRS2. (*A*) MRS2 displays no significant hit against any Pfam domain and contains as sole features a central coiled-coil domain and two transmembrane domains. (*B*) The phylogenetic profile of MRS2 reveals the existence of orthologs across the entire eukaryotic kingdoms despite a predicted low traceability. The presence of an ortholog in a given species is indicated by a dot. The cell color represents protein traceability. (*C*) Section of the MRS2 alignment considering orthologs from different representatives across the eukaryotic tree of life. The selected region shows exemplarily for the entire alignment that MRS2 orthologs share conserved sequence motifs that most likely are associated with the functionality of this protein as an Mg2+ membrane transporter. As these conserved domains are not represented in a Pfam domain, protTrace cannot consider the corresponding evolutionary constraints during its simulation.

The integration of traceability and ortholog search for the yeast proteins reveals that we sometimes (5%) detect an ortholog although the traceability index of the seed protein predicts that we should not. Reducing the traceability cutoff has little effect on this number (fig. 2*B*). Reasons that explain the discrepancy between the traceability index estimate and the outcome of an ortholog search are diverse. On the one hand, overestimates of the protein-specific evolutionary rates can artificially decrease the traceabilities—although protTrace is considerably robust with respect to variation in the rate estimates (supplementary fig. S13, Supplementary Material online). On the other hand, spurious ortholog assignments can mimic the presence of an ortholog, an artifact that is obviously hard to control (supplementary text, Supplementary Material online). One main—but not the only (supplementary text, Supplementary Material online)—factor determining a protein’s traceability, however, is its Pfam domain content (Finn et al. 2016), as protTrace exploits the characteristic sequence features of Pfam domains to deduce constraints on the evolutionary process (Koestler et al. 2012). In the yeast data, 1,255 out of 6,352 proteins do not have Pfam domains. In the simulated sequence evolution, these proteins evolve without position-specific constraint, and correspondingly many have overall low traceability indices (supplementary fig. S9, Supplementary Material online). This implies that protTrace, if information concerning local constraints on the sequence-specific evolutionary process is not available, can underestimate the traceability of a protein. Figure 3 describes an illustrative example. The yeast protein MRS2 is a mitochondrial inner membrane Mg^2+^ transporter (Wiesenberger et al. 1992), and its traceability indices in species outside fungi are substantially below the threshold of 0.75 (supplementary table S2, Supplementary Material online). The overall low traceability estimate coincides with the absence of Pfam domains in the MRS2 sequence (fig. 3*A*). However, we find yeast MRS2 orthologs across the entire eukaryotic domain (fig. 3*B*), indicating that protTrace underestimates the traceability in this case. An MSA of these orthologs resolves the apparent discrepancy (fig. 3*C*). MRS2 harbors evolutionarily highly conserved domains, which do not occur in Pfam, and thus could not be taken into account during the traceability estimation. Notably, when we generate a custom pHMM from the MRS2 alignment and use this as a constraint model for the sequence simulation within protTrace, the mean traceability of this protein increases from 0.07 to 0.97 (data not shown). Thus, it is crucial for a correct estimate of TI to have full feature information about a protein, which will increase in the future. Within 2 years, the number of Pfam models increased from 14,831 (release 27, Finn et al. 2014) to 16,295 (release 29, Finn et al. 2016). It might be interesting to note that discrepancies between traceability and evolutionary profile, as exemplified by MRS2, can be easily applied to automatically screen for further such instances, where a functional domain is currently not described in Pfam. In these cases, it is then advisable to start protTrace with the option to extract site-specific constraints on the evolutionary process directly from an MSA of orthologs, similar to previous approaches (Alba and Castresana 2007; Moyers and Zhang 2015, 2016).

### Traceability and Subcellular Localization Are Linked

**Fig. 4.**
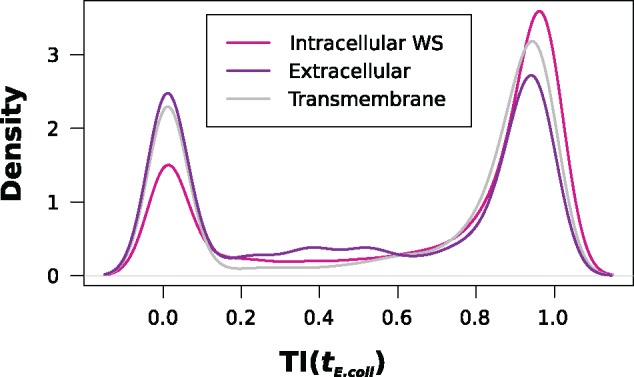
—Density plot of the TI(*E. coli*) for yeast proteins in dependence of their subcellular localization. Water-soluble intracellular proteins tend to have higher traceability indices in *E. coli* compared with proteins with a predicted extracellular localization, and to proteins localized in the cell membrane.

Protein traceability informs whether or not the sensitivity of an ortholog search is sufficient to accurately determine the phylogenetic profile of a protein even in distantly related species. Initial evidence that this measure can provide an alternative view on the interpretation of conservation patterns of orthologs across species comes from the analysis of proteins with different subcellular localization. It was reported that extracellular proteins and, to a somewhat lesser extent, transmembrane proteins have higher evolutionary rates when compared with intracellular proteins (Julenius and Pedersen 2006; Cui et al. 2009; Liao et al. 2010). To see whether this is reflected in differences in the evolutionary traceability, we performed a GO term enrichment analysis in the set of yeast proteins with a TI(*t_E.coli_*) < 0.75, using the cellular component ontology. This revealed that proteins annotated with the GO terms *cell wall*, *anchored component of membrane*, and *extracellular region* are significantly enriched in this set (supplementary fig. S15, Supplementary Material online). Subsequently, we classified the yeast proteins into three groups—membrane proteins, extracellular proteins, and intracellular proteins. We then plotted the TI(*t_E.coli_*) distribution for each of the three protein sets (fig. 4). In line with the findings from the GO enrichment analysis, we find that a greater fraction of proteins with a predicted extracellular localization have a TI(*t_E.coli_*) < 0.75 than is the case for intracellular proteins. Proteins predicted to be anchored to the cell membrane show an intermediate pattern. In light of these results, we expect that an ortholog search is prone to more often miss a distantly related ortholog for extracellular and membrane proteins than for intracellular proteins. This is in line with a recent observation that extracellular proteins have sparser phylogenetic profiles and fewer detected orthologs than intracellular proteins (Sojo et al. 2016). The authors of this study provided two alternative explanations for this difference in size and taxonomic composition of the ortholog groups. A rapid evolutionary turnover, particularly in the case of membrane and extracellular proteins, that is, their replacement by nonorthologous proteins, is a result of ecological niche adaptation. It results in smaller ortholog groups. Alternatively, the higher evolutionary rate of membrane and extracellular proteins could interfere with the remote ortholog identification. Although they provided empirical examples in support of the “turnover hypothesis,” they did not show in which cases the higher evolutionary rate becomes a limiting factor. The evolutionary traceability of a protein, introduced by us, facilitates a more differentiated view. We can now identify such proteins—together with the critical evolutionary distance—for which an ortholog identification is likely to fail due to sensitivity issues, and those where sensitivity is not an issue.

### Protein Traceability, Molecular Function, and Gene Age Estimates Are Linked

Earlier studies have reported the rapid evolution of proteins that are part of the immune defense, reproductive processes, cell adhesion, and transmembrane transport (Swanson and Vacquier 2002; Panhuis et al. 2006; Voolstra et al. 2011). For the yeast example, we evaluated the link between the traceability of a protein and its function, as represented in the assignment of GO terms (Ashburner et al. 2000). We split the 6,352 yeast proteins into three bins based on their TIs in *E. coli* (TI(*t_E.coli_*) ≥ 0.75: 3,947 proteins; 0.75 > TI(*t_E.coli_*) ≥ 0.25: 742 proteins; TI(*t_E.coli_*) < 0.25: 1,663 proteins). A subsequent characterization with GOrilla (Eden et al. 2007, 2009) and visualization of the results with Revigo (Supek et al. 2011) reveal that GO terms are not identically distributed across the three categories (supplementary fig. S10, Supplementary Material online). The 3,947 high-traceability yeast proteins (TI(*t_E.coli_*) ≥ 0.75) are significantly enriched for catalytic functions (supplementary fig. S16*A*, Supplementary Material online). Among these, we find 98% of the 980 yeast enzymes annotated by the Enzyme Commission (EC). Regulatory functions, in turn, are overrepresented in the group of 742 proteins with intermediate traceability indices between 0.75 and 0.25 (supplementary fig. S16*B*, Supplementary Material online). The proteins with a traceability index in *E. coli* below 0.25 are preferentially involved in cell aggregation and cell reproduction (supplementary fig. S16*C*, Supplementary Material online). Altogether, we find that 17% of essential proteins (Giaever et al. 2002) and 70% of the yeast transcription factors have a TI below 0.75 in *E. coli* (supplementary table S3, Supplementary Material online). The low traceability implies that the orthology between regulatory proteins, as well as between proteins of other essential functionalities, is difficult to detect across distantly related species. Consequently, such functions should be underrepresented in the reconstructions of ancient gene sets, not because they are necessarily evolutionary younger, but because information about their evolutionary ancestry decays rapidly.

The 1,203 yeast proteins that are represented in the reconstructed gene set of LUCA (Goldman et al. 2013) exactly match this prediction. They are almost exclusively (96%) recruited from the high-traceability bin. They comprise about half (47%) of all EC annotated yeast enzymes, but merely 4% of the 245 transcription factors with a known binding site (de Boer and Hughes 2012). When taken at face value, this observation translates into a complex evolutionary scenario: The molecular “hardware” of contemporary species, consisting mainly of enzymes, ion transporters, and proteins involved in ribonucleoprotein complex assembly, was largely already established first in LUCA. The regulatory “software” controlling the transcription of genes, however, was either independently rebuilt or invented multiple times in individual evolutionary lineages (Charoensawan et al. 2010). In light of the limited traceability of proteins involved in regulation, it is worth considering a second, more parsimonious explanation. In addition to enzymatic activity, other essential functions might have had a unique genesis early in organismal evolution. However, because rate and pattern of evolutionary sequence change for some of these proteins has eradicated all traces of their ancient origins, it appears as multiple independent inventions of the same function on individual evolutionary lineages.

### Evolutionary Traceability of the Bacterial MG Set Syn3.0

**Fig. 5.**
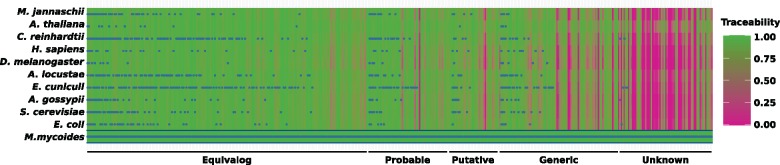
—Phylogenetic distribution and traceability profile for the Syn3.0 minimal gene set. The background color gives the information of the traceability index. The categorization according to the functional annotation status of the individual proteins was adapted from Hutchison et al. (2016).

A reanalysis of the data generated by the Artificial Life Project (Hutchison et al. 2016) corroborates the findings from the previous section. The artificial life project synthesized a self-replicating bacterium (Syn3.0) on the basis of only 438 protein-coding genes from the bacterium *M.mycoides* (Hutchison et al. 2016) (MG). This collection of essential genes comes close to what Koonin (2003) referred to as an absolute MG set, that is, the set of genes that an organism requires under the most optimal conditions. One could naively assume that many of these genes are essential for cellular life in general, and are thus conserved across the tree of life. As a consequence, they should be represented in the gene set assigned to LUCA. To assess the phylogenetic distribution of the 438 genes, we replaced the unidirectional BLAST search performed by Hutchison et al. (2016), which does not inform about the precise evolutionary relationships of the identified homologs, with an ortholog search (fig. 5 and supplementary table S4, Supplementary Material online). This revealed that 170 of these genes have no detectable ortholog outside *Mycoplasma*, and for 149 genes the exact biological function is unclear. On the first sight this might imply that *Mycoplasma* has evolved its own path to organismal functionality, reflecting that a set of genes essential for one species may not be essential for another organism (Gerdes et al. 2003; Koo et al. 2017). However, we found that 60 proteins in MG have traceability indices below 0.75 in any tested species outside *Mycoplasma*. Among these are the majority of proteins with unknown functions (41/65), and additionally 15 of the 84 proteins with only a generic function assigned (fig. 5). Whatever essential tasks these 60 proteins have, it may be premature to mark them as *Mycoplasma*-specific inventions. Instead, we hypothesize that their low traceability blurs the evolutionary link to related proteins with the same function in other organisms. Given their participation in fundamental cellular functioning, it is tempting to speculate that these proteins can provide relevant hints toward the nature of the “software” that appears missing in the current reconstructions of the LUCA gene set.

### Protein Traceability Limits Ortholog Identification in the Fast-Evolving Microsporidia

**Fig. 6.**
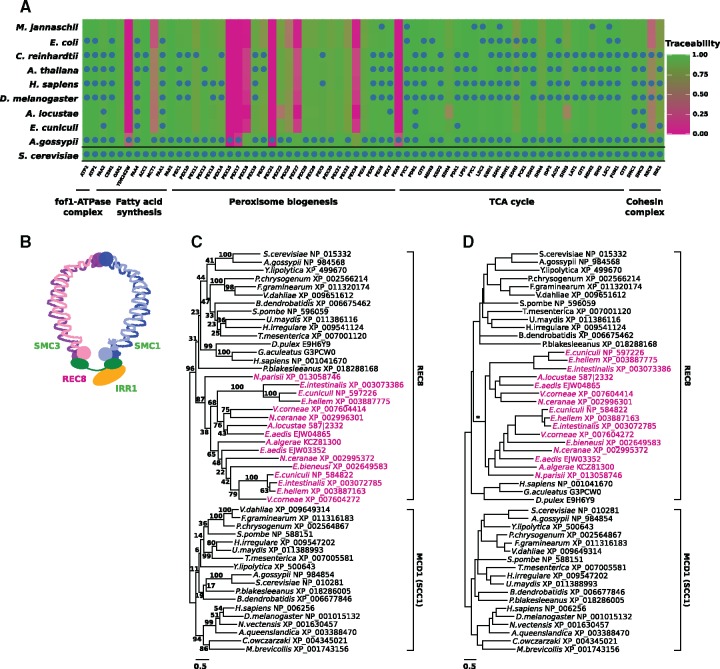
—(*A*) Phylogenetic profiles for the components of fungal key metabolic pathways across ten representative species from the tree of life. The background color gives the information of traceability index ranging from green (high traceability) to red (low traceability). (*B*) The four proteins of the yeast cohesin complex form a ring-like structure. Font color of the protein names indicates that TI(*t*) in the microsporidium *Encephalitozoon cuniculi* is either 0.75 or higher (green), or below (red). (*C*) Maximum likelihood tree of REC8 and MCD1 (syn. SCC1) orthologs. The microsporidian REC8 candidates are colored in red. Branch labels represent percent bootstrap support. (*D*) Alternative phylogeny for the REC8/MCD1 (SCC1) protein family. It features monophyletic fungal REC8 and MCD1 (SCC1), respectively. The animal REC8 proteins are placed as sister to monophyletic fungal and microsporidian REC8 proteins. The branching orders in the fungal subtrees follow the accepted species phylogeny. The alternative tree is with a Δ_LogLikelihood_ = 25.7 not significantly worse than the ML tree shown in (*C*) (Shimodaira–Hasegawa test: *P* > 0.05). The asterisk indicates a gene duplication on the microsporidian lineage that gave rise to the two paralogous microsporidian REC8 lineages.

Microsporidia, intracellular parasites closely related to fungi (Corradi and Keeling 2009) are a hallmark example that a low traceability can result in essential genes being overlooked. All microsporidia analyzed so far share two characteristics: First, their genomes harbor between 2,000 and 4,000 genes, due to an ancient radical reduction in genome size (Slamovits et al. 2004). Second, their genomes, together with the proteins encoded therein, evolve extraordinarily fast. Although the first characteristic makes it tempting to generally equate a nondetection of an ortholog to a yeast protein with a gene loss, the high evolutionary rate of microsporidia indicates that a low traceability may be another reason for the lack of orthologs. Katinka et al. (2001) and Cuomo et al. (2012) showed that key metabolic functions, for example, the fof1-ATPase complex, fatty acid synthesis, the tricarboxylic acid cycle, and the formation of peroxisomes are absent in microsporidia (Katinka et al. 2001; Cuomo et al. 2012). We determined the phylogenetic profiles for the corresponding yeast proteins and could confirm that for many proteins no ortholog was detectable in our microsporidian representatives (fig. 6*A* and supplementary table S5, Supplementary Material online). For most of these proteins, the traceabilities in microsporidia are in the range of 0.9 and above. This indicates that the corresponding genes have been lost on the microsporidian lineage.

The situation is different for proteins involved in meiosis and recombination. Yeast, as well as most other eukaryotes, share a conserved set of 29 proteins involved in these processes (Malik et al. 2007). Microsporidia lack orthologs to six of these proteins (Cuomo et al. 2012) (supplementary table S6, Supplementary Material online). However, for three out of these six cases the traceability of the yeast protein in microsporidia is low. This provides a clear indication that orthologs might have been overlooked. One protein, REC8, exemplifies the problem best. In yeast, REC8 forms with IRR1, SMC1, and SMC3 the cohesin complex, a ring-like structure that keeps sister chromatids connected during meiosis (Klein et al. 1999) (fig. 6*B*). Interestingly, *E. cuniculi* harbors orthologs to three of the four genes (fig. 6*A* and supplementary table S6, Supplementary Material online). This raises the question about the whereabouts of REC8, the fourth member of this complex, which closes the ring-like structure. So far, a single report claims the presence of REC8 in the microsporidium *E. cuniculi* (Malik et al. 2007). However, the search strategy that was used—a unidirectional PSI-BLAST search (Altschul et al. 1997)—lacks the precision to support this conclusion (Chen et al. 2007). Consequently, a study based on ortholog searches reported the absence of this protein in *E. cuniculi*, and it identified *N. parisii* as the only microsporidian species harboring an ortholog to the fungal REC8 (Cuomo et al. 2012). To explain the sporadic presence of REC8 among microsporidia, Cuomo et al. (2012) hypothesized that the shorter period of time that *N. parisii* has been passaged in a laboratory setting, compared with other microsporidian species, caused the retention of REC8 only in this species. To resolve the controversy, we consulted the traceability of REC8 (fig. 6*A*). With a value of 0.5, the traceability index in *E. cuniculi* is substantially below the empirically determined threshold of 0.75. We took this as a reason for increasing the search sensitivity to identify highly diverged microsporidian REC8 orthologs, taking, however, the risk to end up with false positive predictions. In the first step, we screened the protein sets of ten microsporidian species for sequences harboring the Rad21_Rec8_N Pfam domain (PF04824), which occurs in REC8. This identified in six of the 11 species two proteins each, among them *E. cuniculi.* In each of the remaining four species, only a single protein carried the PF04824 domain, among them *N. parisii*. We then extended the search to other eukaryotes (supplementary fig. S17, Supplementary Material online). Fungi, in general, possess two proteins with the PF04824 domain. In yeast, these correspond to REC8 and MCD1 (synonym SCC1). MCD1 is the protein that replaces REC8 in the cohesin complex during mitosis (Klein et al. 1999). Thus, the identification of two microsporidian proteins with the Rad21_Rec8_N domains resembles the situation generally seen in fungi. However, at this step of the analysis, the precise identity of the microsporidian proteins remains unclear.

In the next step, we reconstructed the evolutionary relationships of a subset of fungal and nonfungal REC8 and MCD1 (SCC1) orthologs together with the microsporidian candidates (fig. 6*C*). Although this tree is not well resolved and renders, for example, the fungal REC8 proteins paraphyletic, it already supports a grouping of the microsporidian sequences with fungal and animal REC8 orthologs. Subsequently, we rearranged the tree topology to reflect the accepted evolutionary relationships of fungi, microsporidia, and animals. A topology test revealed that the likelihood of the rearranged tree is with a Δ_LogLikelihood_ = 25.7 not significantly worse than the ML tree ( Shimodaira–Hasegawa test: P < 0.05; Shimodaira and Hasegawa [1999]). The data are therefore compatible with the hypothesis that microsporidian REC8 candidates form the sister clade of the fungal REC8 proteins, to the exclusion of the animal REC8 proteins (fig. 6*D*). Paired with the observation that the domain architecture of the microsporidian proteins agrees with that of yeast REC8 (supplementary fig. S18, Supplementary Material online), this indicates that we have indeed identified the missing REC8 orthologs in microsporidia.

In summary, the REC8 example shows that missing orthologs in the quickly evolving microsporidia are not exclusively an effect of the rampant gene loss that is characteristic for the group (Corradi and Slamovits 2011). Here, we provide for the first time convincing evidence that REC8 orthologs are widespread among microsporidia. The meiotic cohesin complex might therefore function in microsporidia as described for yeast. It should be noted, however, that we find no trace of MCD1 (SCC1), the mitotic counterpart of REC8. As this protein has a high traceability in the microsporidia, we propose a genuine gene loss of the Mcd1 gene (supplementary table S6, Supplementary Material online). In this context, it is intriguing that we observe two paralogous REC8 proteins in the microsporidia, whose emergence via a gene duplication can be dated to the last common ancestor of the microsporidia. Notably, six out of ten microsporidian species harbor both paralogs. It is tempting to speculate that the apparent loss of the *Mcd1* (*Scc1*) gene on the microsporidian lineage was compensated by a duplication of *Rec8*.

## Conclusion

Orthologs form the essential basis to propagate functional annotations between proteins of different species and to reconstruct the evolutionary past. So far, it has largely remained a matter of speculation as to what extent limitations in the sensitivity of ortholog searches have influenced insights gained from these reconstructions. Here, we have presented a software, protTrace, facilitating a simulation-based procedure to assess the evolutionary traceability of a seed protein over time when using standard ortholog searches. In contrast to existing approaches, protTrace infers constraints on the evolutionary sequence change of the seed protein from the presence of Pfam domains. This has two main advantages: The constraint estimates are independent from the availability and the phylogenetic diversity of orthologs to the seed protein; and the constraint pattern for a protein depends only on its Pfam domain composition and not on the species it was derived from. The generally high traceability of enzymes indicates that orthologs are readily identifiable throughout the tree of life, explaining why ancestral gene set reconstructions are enriched for catalytic functions. This is contrasted by proteins involved in regulatory processes, for which traceability implies that most of the signal informing about any ancient evolutionary origin has long been lost. Future attempts to reconstruct the evolutionary history of a protein from its phylogenetic profile will now have the possibility to adapt the sensitivities of ortholog searches according to the traceabilities of the individual proteins. If the traceability of a protein is high, an increase of the search sensitivity—which naturally comes at the cost of a reduced specificity—is bound to result in false positive predictions. However, if the traceability is low, more sensitive searches may detect faint signals of an evolutionary relationship between proteins in two species. In these cases, a careful downstream analysis including domain architecture comparison, phylogenetic tree reconstruction, and screen for interacting partners is then required to validate candidates resulting from such a relaxed search. For the example of yeast REC8, we demonstrated that a limited traceability is indeed an issue that compromises ortholog detection and can lead to wrong evolutionary conclusions. Contrary to current belief, we could show that REC8 is present and widespread in microsporidia, rendering the cohesin complex complete and probably functional. Thus, microsporidia bring along the necessary prerequisite for both meiosis and recombination.

In summary, the evolutionary traceability of proteins brings us one step closer toward deciding when the absence of evidence for an ortholog is evidence for its absence and when it is not (Alderson 2004).

## Supplementary Material

Supplementary data are available at *Genome Biology and Evolution* online.

## Supplementary Material

Supplementary Data
