# Supplementary material for: The Evolutionary Traceability of a Protein
Source: Genome Biol Evol. 2019 Jan 15;11(2):531–45. doi: 10.1093/gbe/evz008 (PMC6394115; doi:10.1093/gbe/evz008)
Supplement: Supplementary Data [file evz008_supp.zip › supplementary_materials.GBE.r1-submit.docx]

Supplementary material

Supplementary Text 2

Sensitivity and specificity of the ortholog search tool 2

Pfam domain content is not the sole determinant of protein traceability. 2

Supplementary Tables 4

Supplementary Figures 5

Fig. S1. – The workflow of protTrace. 5

Fig. S2. – Comparison of indel rate estimates using Sparta and the parsimony algorithm of protTrace 6

Fig. S3. – Distribution of evolutionary parameter estimates across the yeast gene set. 7

Fig. S4. – Tree view of the traceability indices of yeast MSR2 across the 232 target taxa. 8

Fig. S5. – Distribution of the ortholog group sizes for the yeast protein set. 9

Fig. S6. – Correlation between the evolutionary rate estimates and the ortholog group size. 9

Fig. S7. – Influence of variations in the indel length distribution on the Traceability Index calculation. 10

Fig. S8. – Mean traceability indices for the proteins with default scaling factor and default indel rate. 11

Fig. S9. – Dependency of the traceability index estimate on the choice of the ortholog detection program. 12

Fig. S10. – Mean traceabilities of the yeast protein set based on different training data. 12

Fig. S11. – Influence of the training data on the site-specific rate scaling factor estimation. 14

Fig. S12. – Influence of the simulation procedure on the traceability estimation. 15

Fig. S13. – Effect of scaling factor and insertion/deletion rate variation on the traceability index estimates. 16

Fig. S14. – Pfam domain content influences protein traceability. 17

Fig. S15 - Gene Ontology term enrichment (cellular component ontology) in protein with a low traceability. 18

Fig. S16. – Gene Ontology term enrichment (Biological Process) in protein sets with different traceabilities. 19

Fig. S17. – Number of protein sequences harboring a Rad21_Rec8_N domain. 20

Fig. S18. – Pfam Domain architecture evolution in the REC8 and MCD1 gene families. 21

Accessory data 22

References 22

# Supplementary Text

Sensitivity and specificity of the ortholog search tool

Spurious ortholog assignments can be a further reason for incongruences between traceability of a protein and of its phyletic distribution. A recent benchmark has again revealed that so far, no ortholog assignment tool is error free, and individual approaches differ in both sensitivity and specificity (Altenhoff, et al. 2016). Obviously, both will have an effect on whether or not an ortholog is detected for a seed protein with a given traceability. For example, our results slightly change, when we switch the ortholog search procedure for the 6,352 yeast proteins. Using the OMA-based (Roth, et al. 2008) ortholog search, we detect in about 5% of the cases a eukaryotic ortholog despite a predicted traceability of below 0.75. If we repeat the same analysis, this time determining the phylogenetic profiles across eukaryotes with OrthoDb (Zdobnov, et al. 2017), for which the authors claim a higher sensitivity, the fraction of identified eukaryotic orthologs with traceability below 0.75 increases slightly to 7%. In such instances, only a case-by-case assessment of whether or not the additionally identified candidates indeed represent genuine orthologs can resolve the issue (see supplementary figure S9).

Pfam domain content is not the sole determinant of protein traceability. Individual proteins have traceability indices close to one across the entire tree of life despite the absence of Pfam domains (see supplementary fig. S14). In turn, examples abound where the traceability is low although at least one Pfam domain could be annotated in the sequence. This highlights that other factors such as protein specific substitution rates reflected in the scaling factor κ, and also protein specific indels rates influence protein traceability. We therefore explored the dependency of protein traceability on protein-specific evolutionary rate captured in κ, and on the indels rates, respectively. We grouped the yeast proteins into four bins according to TI(*t_E. coli_)*: (i) <0.25, (ii) 0.25 – 0.5, (iii) 0.5 – 0.75, and (iv) >0.75. We then selected from each bin randomly 25 yeast proteins. For each of these 100 proteins, we subsequently doubled and halved its κ, respectively, and assessed the effect on the protein’s traceability. Likewise, we changed the indels rates by a factor of 10 and 0.1. The results are shown in supplementary fig. S13. Note, that a change of the indels rates by an order of magnitude was necessary to observe a noticeable effect in the mean traceabilities. Supplementary fig. S13 shows that the traceability is negatively correlated with both rates, however the change of κ has a substantially stronger effect. The figure, however, also suggest that only slight changes of the evolutionary parameters, as they may be caused by the variance of the evolutionary parameter estimates should not have a severe effect on the traceability estimates.

# Supplementary Tables

Supplementary table S1 | List of 232 representative species from the three domains of life

Supplementary table S2 | Traceability indices of 6352 S. cerevisiae proteins in 232 representative species

Supplementary table S3 | Traceability indices of yeast proteins in *E. coli* and classification into essential genes and the LUCA genes

Supplementary table S4 | Traceability analysis of the Mycoplasma mycoides genes representing the minimal gene set for a self-replicating cell (Syn3.0)

Supplementary table S5 | Phylogenetic profile and traceability indices of yeast proteins involved into core metabolic pathways in microsporidia

Supplementary table S6 | Phylogenetic profile and traceability indices of yeast meiotic proteins in microsporidia

# Supplementary Figures

Fig. S1. – The workflow of protTrace. **A**, Overview of the individual steps to assess the evolutionary traceability, TI*(t)*, of a protein. The procedure is described in full detail in the Results section of the main text. **B**, Maximum parsimony based approach to estimate insertions / deletions (indels) rates and length distribution parameters. We split the MSA whenever a gap starts. Subsequently, we construct a transformed alignment by counting the gaps (if any) for every sequence in each split alignment part. We then calculate the maximum parsimony score for each column of the transformed alignment given the tree inferred earlier from the original alignment. Here, the maximum parsimony score is the number of insertions and deletions required to obtain the transformed alignment. Insertion and deletion rates per position, respectively, are then obtained by dividing the half of the number of events by the product of the tree length and the alignment length. The insertion and deletion lengths of one most parsimonious solution are used to infer *p*, the parameter for the geometric length distribution.


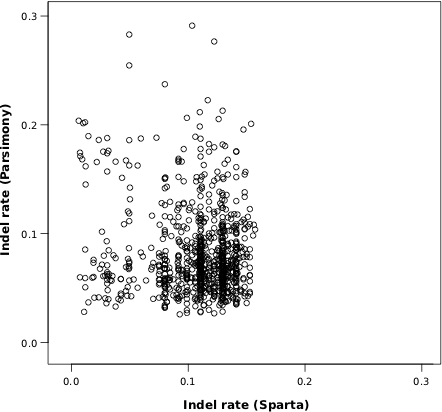


Fig. S2. – Comparison of indel rate estimates using Sparta and the parsimony algorithm of protTrace. The plot shows that indel rates calculated with Sparta (Levy Karin, et al. 2015) and with the parsimony approach implemented into protTrace are in the same range, with a tendency of the parsimony based approach to result in slightly lower indel rates. Note, for the maximal upper indel rate of Sparta we kept the default value of 0.15.


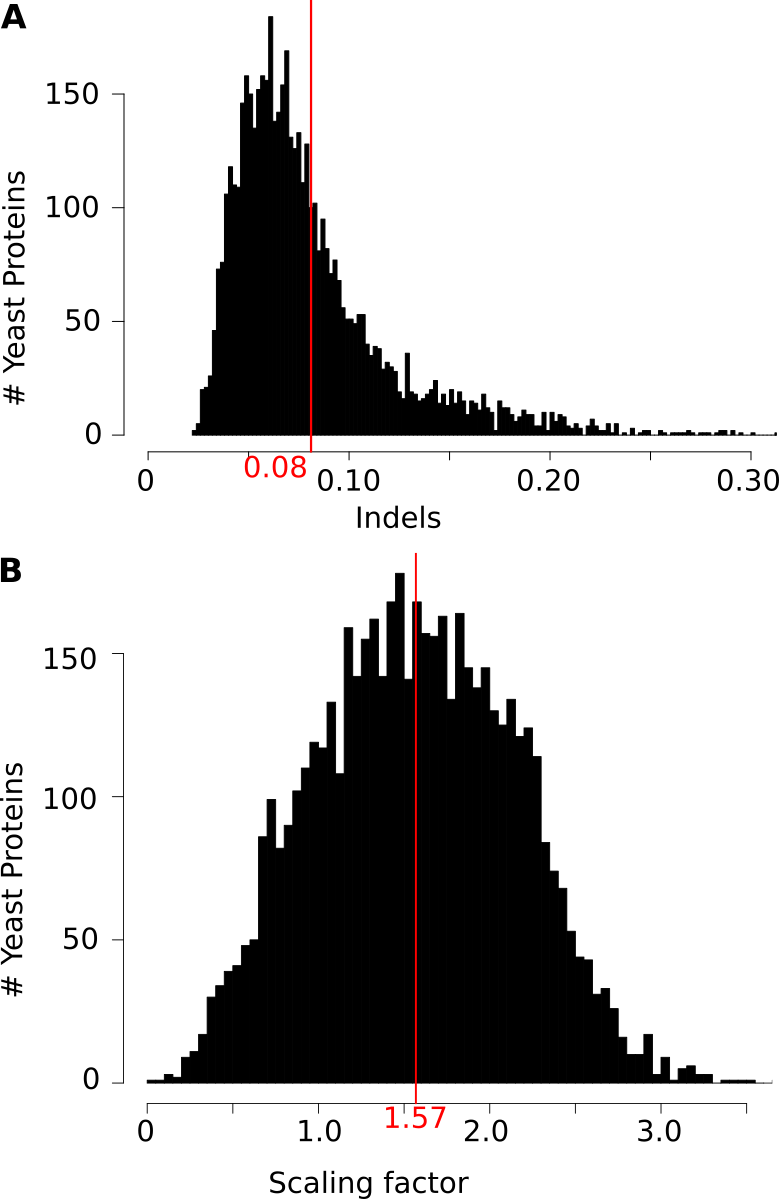


Fig. S3. – Distribution of evolutionary parameter estimates across the yeast gene set. **A**, The histogram shows the distribution of the insertion/deletion (indel) rates estimated for all yeast proteins having at least three orthologs. The mean value is indicated in red. **B**, The histogram shows the distribution of the scaling factor κ_seed_ for all yeast proteins having at least two orthologs. The mean is indicated in red.

Fig. S4. – Tree view of the traceability indices of yeast MSR2 across the 232 target taxa. The black arrow indicates the position of *Saccharomyces cerevisiae,* the species the seed-protein was derived from. Green taxon labels indicate a high, yellow an intermediate, and red a low traceability index of yeast MSR2 in the respective species. The cladogram was rooted with *S. cerevisiae*.

Fig. S5. – Distribution of the ortholog group sizes for the yeast protein set. Of the 6,538 proteins in the yeast gene set, 1,271 have fewer than 3 orthologs. Because we require at least four proteins in an ortholog group to empirically estimate the indel rate, we use, for these proteins, the default indel rate of 0.08 for the simulation. For 1,101 proteins, less than 2 orthologs were found. For these proteins, we additionally used a default scaling factor of 1.57.


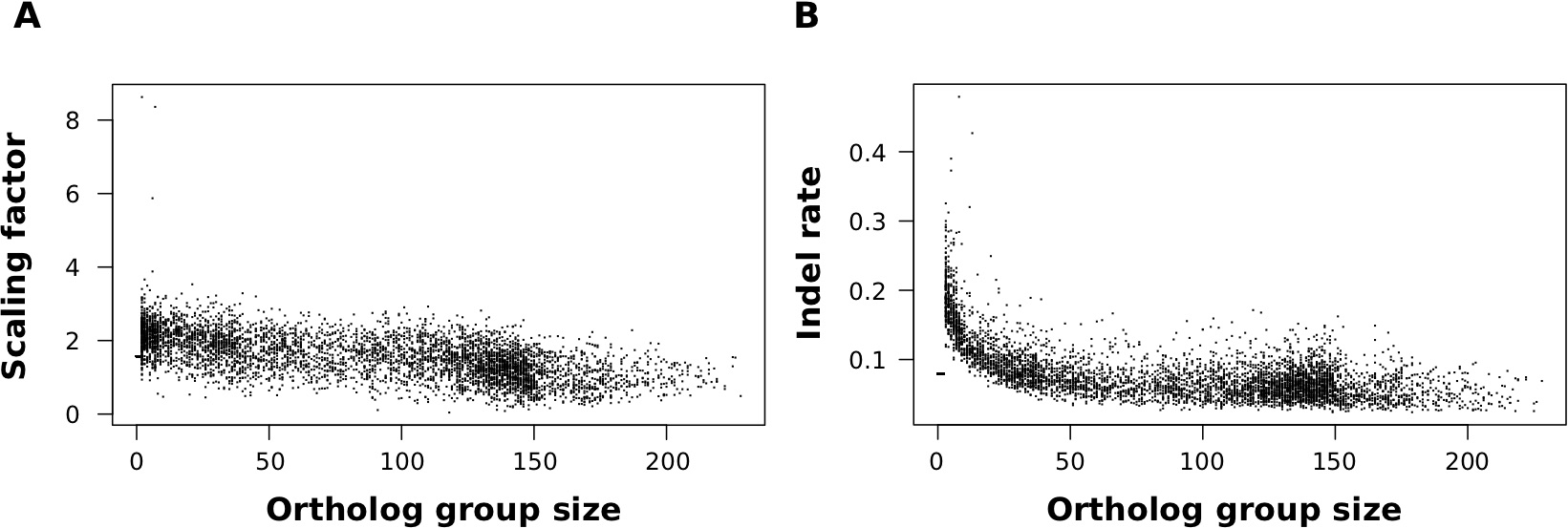


Fig. S6. – Correlation between the evolutionary rate estimates and the ortholog group size. The plots show that both scaling factor estimates (A) and indel rate estimates (B) are negatively correlated with the ortholog group size. This indicates that methodological issues, such as difficulties in aligning distantly related sequences in large ortholog groups, have no obvious effect on the parameter estimation.


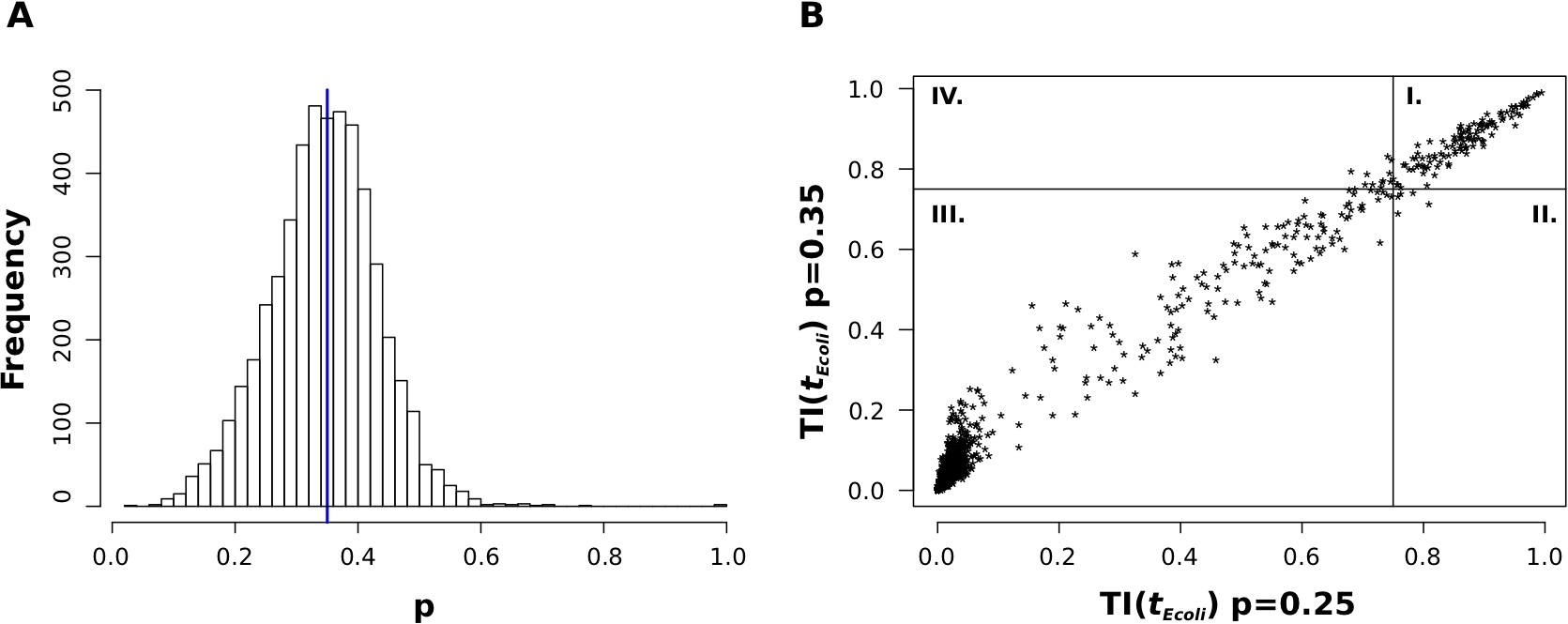


Fig. S7. – Influence of variations in the indel length distribution on the Traceability Index calculation. In the scope of protTrace, REvolver uses two approaches to determines the length of insertions and deletions. In regions that represent a Pfam domain, the insertion and deletion process is parameterized in a site-specific manner using the corresponding profile Hidden Markov Model (see Koestler, et al. (2012)) for further details). Note, this parameterization is independent of the ortholog group size. In regions outside a Pfam domain, the length of insertions and deletions is drawn from a geometric distribution parameterized with *p*, the probability that an indel ends. If an ortholog group has four or more members, we estimate *p* from the data (see supplementary Figure S1B). Otherwise, we use *p=0.25*, which is implemented as the default value into REvolver. **A** shows the distribution of empirical *p* estimates across the 5,081 yeast proteins. The mean of this distribution is with 0.35 higher than the default p used by us. To assess the effect of this deviation, we computed TI(*t_Ecoli_*) estimates for the proteins with less than three orthologs once with p=0.25, and once with p=0.35 (**B**). The TI threshold 0.75, which we use to differentiate between proteins of high and of low traceability, respectively (see Figure 2 in the main text), is indicated by the vertical and the horizontal lines. The plot reveals that the two alternative choices for p have overall only a minor impact on the outcome of the traceability analysis. Interestingly, while we see few proteins that get exclusively assigned to the high TI fraction (TI(*t_E.coli_*)) of 0.75 and above) if we increase *p* from 0.25 to 0.35 (Quadrant IV.), the opposite is seen as well (Quadrant II.). This fluctuation in both directions can be best explained by to the stochasticity of the simulation procedure underlying the traceability estimate (see Fig. S1). We, thus, trust that the effect of choosing either p=0.25 or p=0.35 for parameterizing the indel length distribution in protein regions outside Pfam domains is negligible.

Fig. S8. – Mean traceability indices for the proteins with default scaling factor and default indel rate. For 1,101 yeast proteins, less than two orthologs were found. We could, therefore, not empirically assess the protein-specific scaling factor κ_seed_ and the parameters for modelling the indel process. Instead, we used the default values of κ_seed_ = 1.57 and an indel rate of 0.08, respectively (*see* supplementary Figure S3). The density plot shows for the 1,101 proteins the mean traceability index across our collection of 232 species. While most proteins have an overall low traceability, there is a considerable fraction with mean traceability indeces of 0.75 and above. This indicates that the use of the default values for the evolutionary rate estimates does therefore not determine a low traceability.


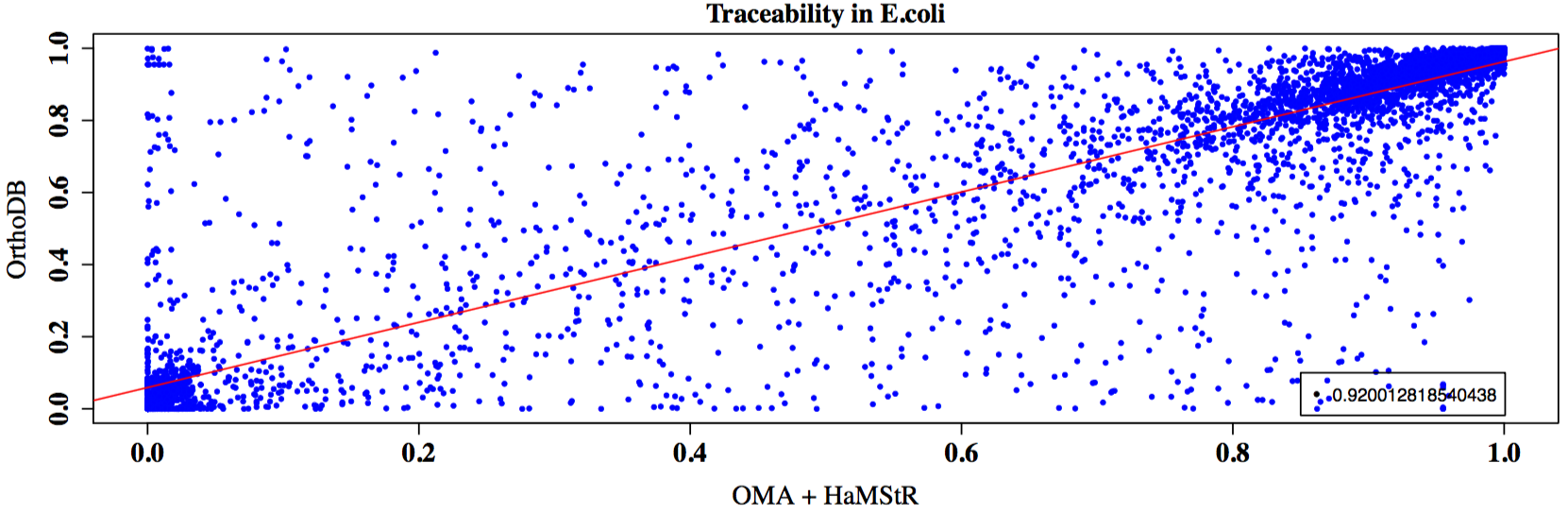


Fig. S9. – Dependency of the traceability index estimate on the choice of the ortholog detection program. We computed the TI(*t_E.coli_*) for each yeast protein once the ortholog groups computed with the Oma+HaMStR combination, or the orthologous groups retrieved from OrthoDB. Both traceability estimates are highly correlated (r = 0.92) indicating that the traceability estimate is considerably robust with respect to the choice of the orthology inference method. In particular, considerably few proteins are of high traceability (TI(*t_E.coli_*) ≥ 0.75) for one dataset, and of low traceability (TI(*t_E.coli_*)<0.75) in the other.

Fig. S10. – Mean traceabilities of the yeast protein set based on different training data. We computed the protein-specific evolutionary parameters for the yeast proteins using orthologs from the full set of 232 species (x axis), and only from fungal species (y axis). The resulting mean traceability estimates are largely unaffected by the difference in diversity of the underlying training data (r = 0.95). This indicates that the phylogenetic diversity of the training data has almost no impact on the traceability estimates for the yeast proteins.

Fig. S11. – Influence of the training data on the site-specific rate scaling factor estimation. We compiled for the 5,259 yeast proteins analyzed by Moyers and Zhang (2016) the training data as described in the original publication. We then estimated the relative rates per site with TreePuzzle using a discrete Γ distribution with 16 rate categories, again in analogy to Moyers and Zhang (2016). The plot shows for each alignment the fraction of sites with a relative rate of 0 (red dots). We then repeated the analysis for the same yeast proteins, this time using an alignment of a phylogenetically diverse set of fungal orthologs to infer the site specific rates (blue dots). The analysis reveals a substantially influence of the composition of the training data on the estimation of the site rates. The use of the evolutionary closely related set of sensu stricto yeast orthologs for inferring the constraints results in a substantial fraction of positions with relative rates of 0. Such positions will remain constant in the course of simulated evolution, and as a consequence result in a high traceability of the respective protein. If, however, the phylogenetically diverse set of orthologs is used for inferring the relative rates for the same set of sequences, the fraction of constant sites decreases substantially. As a consequence, the sequences are now more free to change in the course of simulated evolution, and their traceability will decrease.

Fig. S12. – Influence of the simulation procedure on the traceability estimation. We compared, exemplarily for the yeast protein ACS1, the influence of the simulation procedure and the constraint information on the outcome of the traceability analysis. Inferring the evolutionary constraints from the yeast sensu stricto set (c.f. Moyers and Zhang (2016)) results in overall high traceabilities over the entire simulation distance for both Rose (Stoye, et al. 1998) and when using this data to infer custom constraints for the REvolver simulation (Koestler, et al. 2012). Changing the data underlying the constraint information to include sequences spanning the full diversity of the fungi causes the traceability estimates for the Rose simulation to decrease substantially. Note that they are still slightly higher compared to using REvolver in combination with constraints inferred automatically from Pfam domains (Finn, et al. 2016). This reflects the conceptual differences between the two simulation procedures. Rose uses the same substitution model across sites, and only allows to modify the substitution rate in a site specific manner. In essence, an amino acid is therefore subjected to the same substitution process, irrespective of its sequence context. The limitations of this concept are extensively discussed in Sjölander, et al. (1996). REvolver, in turn, automatically extracts, for each position covered by a Pfam domain, information from the corresponding pHMM to parameterize a site-specific substitution model. By doing so, it better captures a site-specific substitution process as it is reflected in the aligned training data accounting even for unseen events (Sjölander, et al. 1996). It is easily conceivable that this results in a more thorough exploration of the sequence space in the course of simulated evolution. As a result, the traceability curve decreases more quickly in comparison to the Rose simulation. Note, the simulations underlying this figure have been performed without considering the insertion/deletion process.

average scaling factor obtained from the 4971 scaling factors for yeast proteins with at least 3 orthologs.

Fig. S13. – Effect of scaling factor and insertion/deletion rate variation on the traceability index estimates. We plotted the mean traceability index (TI) across 232 species for 100 yeast proteins (25 each from the TI ranges [0-0.25[, [0.25-0.5[, [0.5-0.75[, and [0.75-1]]. We then computed for each protein the mean TI when doubling or halving either the scaling factor or the indel rate, respectively. The plot reveals that the effect of the rate modification on the mean TI is only moderate for the proteins in the high TI range ([0.75-1]; proteins 75 – 100). A more pronounced effect is seen for the proteins in the lower TI ranges. As a general trend, TI estimates are more sensitive to changes in the scaling factor than to changes in the indel rate.


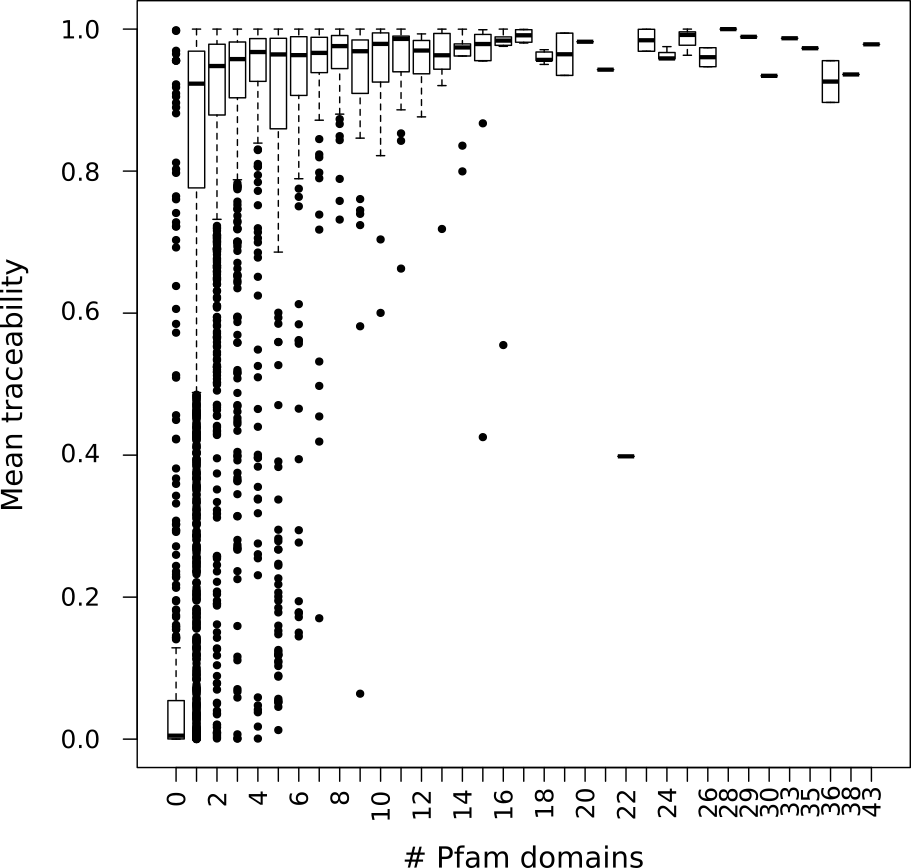


Fig. S14. – Pfam domain content influences protein traceability. The box plot shows the distribution of the mean traceability index across 232 taxa for yeast protein harboring 0 up to 43 Pfam domains. The plot shows that Pfam domain content and protein traceability are overall correlated. However, individual proteins can have high traceabilities even without harboring any Pfam domain. In these cases, low rates for substitutions and indels drive the traceability. In turn, there is a considerable set of proteins with low mean traceability indices despite the presence of Pfam domains. In these cases, the constraints imposed by the pHMM representing the domain are not sufficient to drive local sequence conservation to an extent that it suffices for an ortholog detection over larger evolutionary distances.


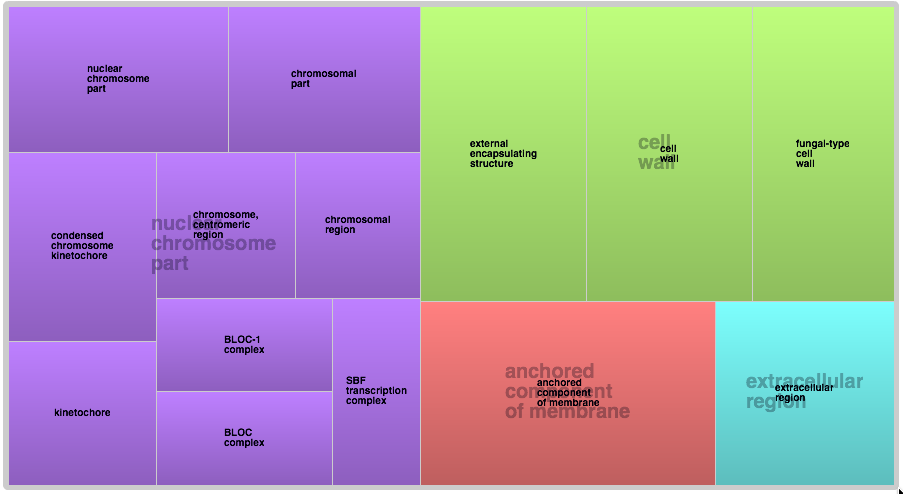


Fig. S15 - Gene Ontology term enrichment (cellular component ontology) in protein with a low traceability. Proteins with a traceability index TI(*E. coli*) <0.75 are enriched for the GO terms “cell wall”, “anchored component of the membrane”, and “extracellular region. The tree maps were generated with REVIGO(Supek, et al. 2011). The underlying data is available from https://figshare.com/projects/yeast_traceability_metadata/56348.


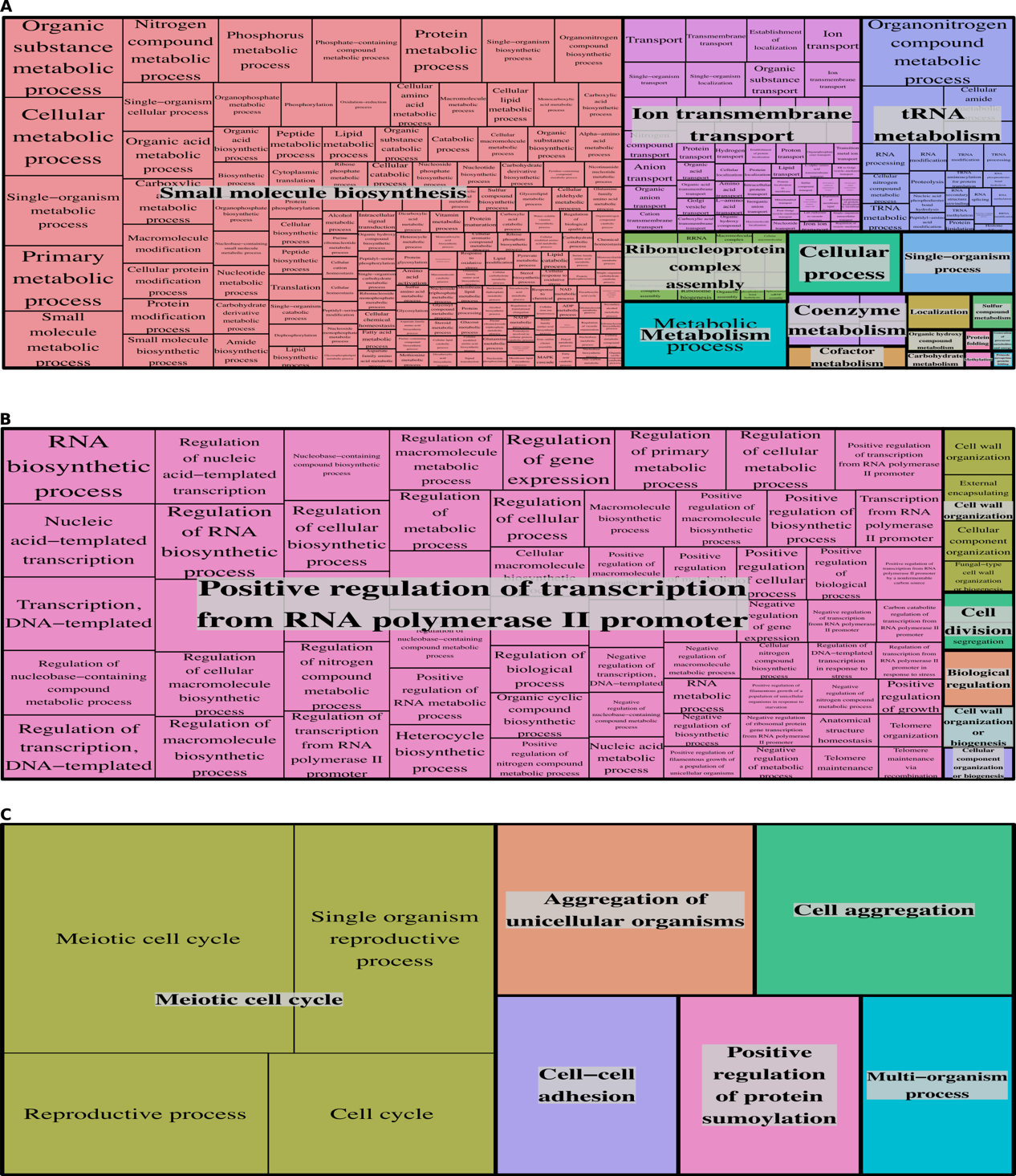


Fig. S16. – Gene Ontology term enrichment (Biological Process) in protein sets with different traceabilities. A, GO enrichment in the high traceability bin (TI(Ecoli) ≥ 0.75). B, GO enrichment in the intermediate traceability bin (0.25 ≤ TI(Ecoli) < 0.75). C, GO enrichment in the low traceability bin (TI(Ecoli) < 0.25). The tree maps were generated with REVIGO(Supek, et al. 2011). The underlying data is available from https://figshare.com/projects/yeast_traceability_metadata/56348.


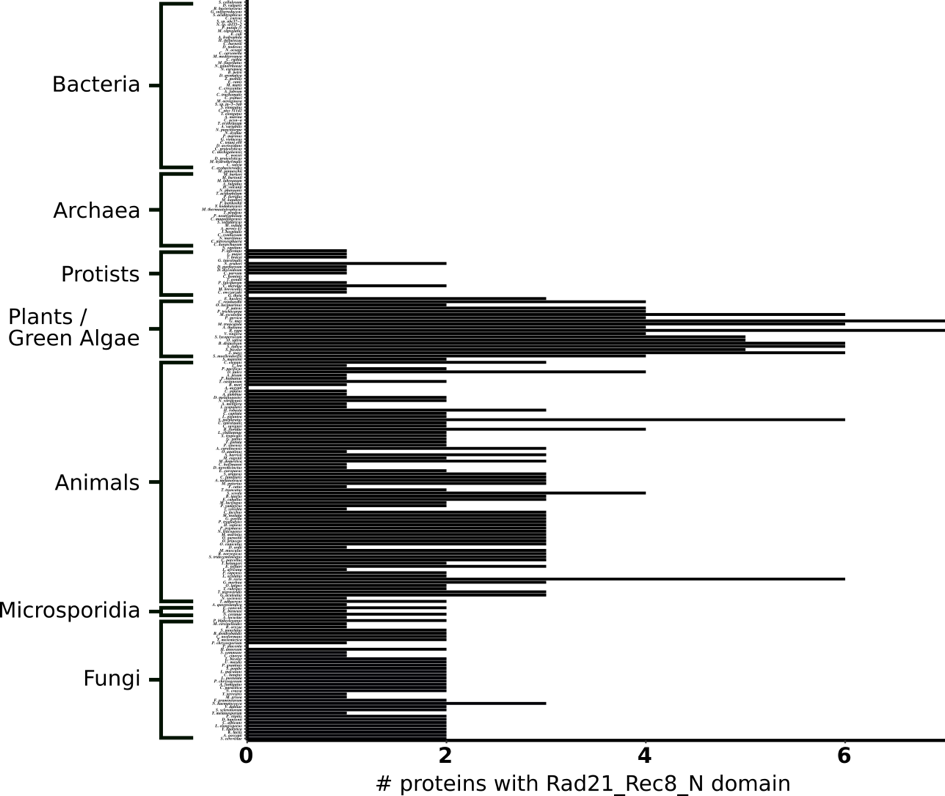


Fig. S17. – Number of protein sequences harboring a Rad21_Rec8_N domain. Fungi, microsporidia and animals mostly possess two proteins with this domain. In Plants, four or more proteins are common, which may be a result of whole genome duplications that occurred on the plant lineage. The Rad21_Rec8 domain appears to be absent in prokaryotes.

Fig. S18. – Pfam Domain architecture evolution in the REC8 and MCD1 gene families. With the exception of two microsporidian proteins, all REC8 and MCD1 proteins share the presence of the Rad21_Rec8_N domain (PF04825) the N-terminus (blue domain). The Rad21_Rec8 domains (PF04824) at the C-terminus of the proteins shows a more diverse presence-absence pattern. All fungal and animal MCD1 (SCC1) proteins share the presence of this domain (shown in green). Within the REC8 clade, the presence of this domain is widespread, however it appears to have been lost twice independently. All microsporidian REC8 proteins (red clade) lack this domain. This indicates a domain loss in the last common ancestor of the microsporidia, and presumably prior to the gene duplication that gave rise to the two paralogous REC8 lineages within the microsporidia (indicated by the asterisk). With that, the microsporidian REC8 proteins resemble the domain architecture of the Sacharomycotina (*S. cerevisiae*, *A. gossypii*, *Y. lipolytica)* and of the Pezizomycotina (*P. chrysogenum, F. graminearum*, *V. dahliae*), which appear to have lost the C-terminal Rad21_Rec8 domain in their last common ancestor.

# Accessory data

All data in support of our analyses is provided via the following link: https://figshare.com/projects/yeast_traceability_metadata/56348. The following information is provided:

- Yeast traceability metadata
  - REvolver input files (indel rates, indel length distribution parameter, and scaling factors)
  - Orthologous groups
  - Yeast protein sequences
- Classification of yeast genes based on their traceability index in *E.coli*
- Classification of yeast genes based on their subcellular localization
  - Membrane proteins
  - Extracellular proteins
  - Intracellular water-soluble proteins
- Syn3 analysis: Data for display in PhyloProfile (Tran, et al. 2018)
- GO-Analysis metadata

# References

Altenhoff AM, Boeckmann B, Capella-Gutierrez S, Dalquen DA, DeLuca T, Forslund K, Huerta-Cepas J, Linard B, Pereira C, Pryszcz LP, et al. 2016. Standardized benchmarking in the quest for orthologs. Nat Methods 13:425-430.

Finn RD, Coggill P, Eberhardt RY, Eddy SR, Mistry J, Mitchell AL, Potter SC, Punta M, Qureshi M, Sangrador-Vegas A, et al. 2016. The Pfam protein families database: towards a more sustainable future. Nucleic Acids Res 44:D279-285.

Koestler T, von Haeseler A, Ebersberger I. 2012. REvolver: Modeling Sequence Evolution under Domain Constraints. Mol Biol Evol 29:2133-2145.

Levy Karin E, Rabin A, Ashkenazy H, Shkedy D, Avram O, Cartwright RA, Pupko T. 2015. Inferring Indel Parameters using a Simulation-based Approach. Genome Biol Evol 7:3226-3238.

Moyers BA, Zhang J. 2016. Evaluating Phylostratigraphic Evidence for Widespread De Novo Gene Birth in Genome Evolution. Mol Biol Evol 33:1245-1256.

Roth A, Gonnet G, Dessimoz C. 2008. Algorithm of OMA for large-scale orthology inference. BMC Bioinformatics 9:518.

Sjölander K, Karplus K, Brown M, Hughey R, Krogh A, Mian IS, Haussler D. 1996. Dirichlet mixtures: a method for improved detection of weak but significant protein sequence homology. Computer Applications in the Biosciences: CABIOS 12:327-345.

Stoye J, Evers D, Meyer F. 1998. Rose: generating sequence families. Bioinformatics 14:157-163.

Supek F, Bosnjak M, Skunca N, Smuc T. 2011. REVIGO summarizes and visualizes long lists of gene ontology terms. PLoS ONE 6:e21800.

Tran NV, Greshake Tzovaras B, Ebersberger I. 2018. PhyloProfile: dynamic visualization and exploration of multi-layered phylogenetic profiles. Bioinformatics 34:3041-3043.

Zdobnov EM, Tegenfeldt F, Kuznetsov D, Waterhouse RM, Simao FA, Ioannidis P, Seppey M, Loetscher A, Kriventseva EV. 2017. OrthoDB v9.1: cataloging evolutionary and functional annotations for animal, fungal, plant, archaeal, bacterial and viral orthologs. Nucleic Acids Res 45:D744-D749.
