# Supplementary material for: The Evolutionary Traceability of a Protein
Source: Genome Biol Evol. 2019 Jan 15;11(2):531–45. doi: 10.1093/gbe/evz008 (PMC6394115; doi:10.1093/gbe/evz008)
Supplement: Supplementary Data [file evz008_supp.zip › SupplementaryTableS2-Info.docx]

**Supplementary table S2** | Traceabilities of 6352 S. cerevisiae proteins in 232 representative species

Due to file size constraints, the table can be accessed online under the following URL:

<https://figshare.com/s/19fbde30d7d9d98d842c>
